# Prognostic Value of the Hemoglobin/Red Cell Distribution Width Ratio in Resected Lung Adenocarcinoma

**DOI:** 10.3390/cancers13040710

**Published:** 2021-02-09

**Authors:** Francesco Petrella, Monica Casiraghi, Davide Radice, Andrea Cara, Gabriele Maffeis, Elena Prisciandaro, Stefania Rizzo, Lorenzo Spaggiari

**Affiliations:** 1 Department of Thoracic Surgery, IEO European Institute of Oncology IRCCS, 20141 Milan, Italy; monica.casiraghi@ieo.it (M.C.); andrea.cara@ieo.it (A.C.); gabriele.maffeis@ieo.it (G.M.); elena.prisciandaro@ieo.it (E.P.); lorenzo.spaggiari@unimi.it (L.S.); 2 Department of Oncology and Hemato-Oncology, Università degli Studi di Milano, 20141 Milan, Italy; 3 Department of Biostatistics, IEO European Institute of Oncology IRCCS, 20141 Milan, Italy; davide.radice@ieo.it; 4 Department of Radiology, Ente Ospedaliero Cantonale (EOC) Istituto di Imaging della Svizzera Italiana (IIMSI), 6903 Lugano, Switzerland; stefaniamariarita.rizzo@eoc.ch; 5 Facoltà di Scienze Biomediche, Università della Svizzera italiana, via Buffi 13, 6900 Lugano, Switzerland

**Keywords:** hemoglobin/red blood cell distribution width ratio (HRR), lung adenocarcinoma, disease-free survival

## Abstract

**Simple Summary:**

Low hemoglobin (Hb) values—indicating a condition of anemia—are related to impaired nutrition and immune system status, suggesting reduced tolerance to therapies in oncologic patients. In fact, it has been shown that pre-treatment anemia predicts poor outcomes in many neoplastic diseases. Similarly, red cell distribution width—which is a measure of the size of variation of circulating erythrocytes—has been shown to be closely related to poor prognosis both in cardiovascular and in oncologic diseases. The use of the Hb-to-red cell distribution width (RDW) ratio (HRR)—which merges data coming from the two blood parameters—is a prognostic marker in esophageal squamous cell carcinoma, small cell lung cancer, and several other types of solid tumors, emerging as an independent prognostic factor for overall survival and disease-free survival. The aim of the present study was to investigate the prognostic role of pre-operative HRR in resected-pulmonary adenocarcinoma patients undergoing a multidisciplinary treatment.

**Abstract:**

Background: The ratio of hemoglobin to red cell distribution width (HRR) has been described as an effective prognostic factor in several types of cancer. The aim of this study was to investigate the prognostic role of preoperative HRR in resected-lung-adenocarcinoma patients. Methods: We enrolled 342 consecutive patients. Age, sex, surgical resection, adjuvant treatments, pathological stage, preoperative hemoglobin, red cell distribution width, and their ratio were recorded for each patient. Results: Mean age was 66 years (SD: 9.0). There were 163 females (47.1%); 169 patients (49.4%) had tumors at stage I, 71 (20.8%) at stage II, and 102 (29.8%) at stage III. In total, 318 patients (93.0%) underwent lobectomy, and 24 (7.0%) pneumonectomy. Disease-free survival multivariable analysis disclosed an increased hazard ratio (HR) of relapse for preoperative HRR lower than 1.01 (HR = 2.20, 95%CI: (1.30–3.72), *p* = 0.004), as well as for N1 single-node (HR = 2.55, 95%CI: (1.33–4.90), *p* = 0.005) and multiple-level lymph node involvement compared to N0 for both N1 (HR = 9.16, 95%CI:(3.65–23.0), *p* < 0.001) and N2 (HR = 10.5, 95%CI:(3.44–32.2, *p* < 0.001). Conclusion: Pre-operative HRR is an effective prognostic factor of disease-free survival in resected-lung-adenocarcinoma patients, together with the level of pathologic node involvement.

## 1. Introduction

Low hemoglobin (Hb) values—indicating a condition of anemia—are related to impaired nutrition and immune system status, suggesting reduced tolerance to therapies in oncologic patients [1]. In fact, it has been shown that pre-treatment anemia predicts poor outcomes in many neoplastic diseases [2,3,4]. Similarly, red cell distribution width (RDW)—which is a measure of the size of variation of circulating erythrocytes—has been shown to be closely related to poor prognosis both in cardiovascular and in oncologic diseases [5,6,7,8]. RDW, in fact, has recently emerged as one of the markers potentially implicated in the inflammatory process and in oxidative stress, as well as in endothelial dysfunction in vessel diseases, a group of alterations implicated both in neoplastic and in non-neoplastic pathophysiology [9]. High baseline values of RDW—defined as anisocytosis—correlate with poor outcomes at least for lung cancer [10,11], breast cancer [12], and renal cancer [13] patients. The use of the Hb-to-RDW ratio (HRR)—which merges data coming from the two blood parameters—was first proposed as a prognostic marker in esophageal squamous cell carcinoma patients [4] and later in small cell lung cancer (SCLC) patients [14], emerging as an independent prognostic factor for overall survival (OS) and disease-free survival (DFS). Although clinical guidelines clearly suggest when to offer adjuvant treatments to patients and when to limit post-operative care to a dedicated follow-up program, they also recommend further evaluation of each patient in a multidisciplinary meeting in order to better customize every treatment to each patient’s need [15]. Further prognostic tools may therefore be taken into consideration to optimize the post-operative strategy and to formulate a more accurate prognostic forecast. The aim of the present study was to investigate the prognostic role of preoperative HRR in resected-pulmonary adenocarcinoma patients undergoing multidisciplinary treatment, in terms of both oncologic outcomes and post-operative complications. The HRR, in fact, reflecting nutritional status, immune system efficiency, and inflammatory condition, may affect not only long-term outcomes but even post-operative complications.

## 2. Materials and Methods

This was an observational retrospective study. Data were collected prospectively, entered into our institutional general thoracic database at the point of care, reviewed, and double-checked retrospectively. Three hundred and forty-two consecutive lung adenocarcinoma patients operated in the last two years (from 2018 to 2019) were analyzed. Patients operated before 2018 were not enrolled due to the only recent introduction of RDW in the standard preoperative assessment of our patients. Written informed consent to undergo the procedure and for the use of clinical and imaging data for scientific or educational purposes, or both, was obtained from all patients before the operation; a blank copy of the written informed consent was provided. Operability was assessed by whole-body computed tomography (CT), whole-body fluorodeoxynucleotide positron emission tomography (PET), and invasive staging procedures, including endobronchial ultrasonographic bronchoscopy (EBUS) and transbronchial needle aspiration (TBNA), as appropriate. The functional status was routinely examined by blood gas analysis and spirometry and by lung perfusion scanning and cardiopulmonary exercise testing (CPET) in the case of planned pneumonectomy. Comorbidities were stratified according to an adapted Charlson comorbidity index [16]. Post-operative death was defined as 30-day mortality or longer if mortality occurred during hospitalization. Complications were classified according to the Thoracic Morbidity and Mortality classification system as minor (grade I and II) and major (grade IIIa, grade IIIb, grade IVa, grade IVb, grade V) [17]. Age, sex, smoking status, type of surgical resection, neo-adjuvant and adjuvant treatments, pathological stage, T and N status, (N0, N1a or N1b, N2 single station or N2 multiple stations) tumor size, pre-operative Hb and RDW and their ratio (HRR), neutrophils, lymphocytes, neutrophil- to-lymphocytes ratio, and lactate dehydrogenase (LDH) levels were recorded for each patient. Outpatient follow-up was performed in order to record the date of relapse (if any); early-stage patients received out-patient follow-up twice a year (every six months); locally advanced-stage patients received out-patient follow-up three times per year (every four months).

### Statistical Methods

Patients’ characteristics, treatments, and procedures were summarized either by count and percent or mean and standard deviation (SD) for categorical and continuous variables, respectively. Disease-free survival was defined as the time from the date of surgery to the last follow-up date without any sign of disease; patients alive without disease at the last follow-up date entered the analysis as time-censored observations. Death from any cause before evidence of recurrence entered the univariate and multivariable disease-free survival analysis as a competing event for relapse. Results were tabulated as hazard ratios (HR) with 95% confidence intervals (CI). Only the variables that were significant in the univariate analysis were included in the multivariable analysis, except Hb and RDW because of their collinearity with HRR. Other collinear variables (pT, stage, and pN) were analyzed together with the significant variables in the univariate analysis, using three stratified-by-treatment multivariable models; of the three models, it was decided to keep the one with the lowest Akaike Information Criterion (AIC) as the best explanatory model. A plot of the cumulative incidence functions for relapse by the median of HRR was produced. Cumulative incidence functions were compared by the Gray’s test. Overall survival was defined as the time from the date of surgery to the date of death from any cause. Complication (any, major, and minor) risks according to the median HRR cut-off were estimated using a logistic regression analysis; results were tabulated as odds ratios (OR) with 95% CIs. The median of the follow-up time was calculated by the inverted Kaplan–Meier method. All tests were two-tailed and considered significant at the 5% level. All analyses were done using SAS 9.4 (SAS Institute Inc., N.C., Cary, USA).

## 3. Results

Baseline patients’ characteristics and treatments are summarized in Table 1.

Forty patients (11.4%) had comorbidities. Among these, 20 patients (5.7%) had a previous history of myocardial infarction, 5 patients (1.4%) suffered from diabetes mellitus without end-organ damage, 4 patients (1.1%) suffered from peripheral vascular disease, 3 patients (0.86%) suffered from cerebrovascular disease, 3 patients (0.86%) suffered from connective tissue disease, 2 patients (0.5%) suffered from chronic obstructive pulmonary disease, 2 patients (0.5%) suffered from mild liver disease, and 1 patient (0.2%) suffered from moderate kidney disease. Seventy-five patients (21.6%) had a previous history of neoplastic disease.

Significant risk factors for relapse in univariate analysis were tumor size (HR = 1.21, 95% CI: (1.04–1.40), *p* = 0.01), Hb level (HR = 0.84, 95% CI: (0.72–0.97), *p* = 0.02), RDW (HR = 1.16, 95% CI: (1.05–1.28), *p* = 0.003), and HRR as both a continuous variable (HR = 0.15, 95% CI: (0.05–0.50), *p* = 0.002) and categorized according to its median cut-off (HR = 0.41, 95% CI: (0.25–0.68), *p* < 0.001); pT2 and pT3 stages but not pT4 also showed an increased hazard for relapse compared to pT1, as well as Stages 2A, 2B and 3A, 3B vs. Stage 1A,1B (Table 2).

Lymph node hilar involvement (N1) and ipsilateral lymph node mediastinal involvement (N2) showed a significantly increased hazard for relapse compared to patients without lymph node involvement (N0): HR = 3.69 95% CI: (2.10–6.48), *p* < 0.001 and HR = 2.85 95% CI: (1.55–5.27), *p* < 0.001, respectively. Multiple- vs. single-station involvement was also a significantly risk factor for relapse HR = 2.84, 95% CI: (1.43–5.63), *p* < 0.001 (Table 2). Treatments were also significantly associated with an increased hazard for relapse, though borderline significant (*p* = 0.04) (Table 2). Risk factors that remained significant in the multivariable analysis were HRR for values ≥1.01 vs. values <1.01 (HR = 0.46 95% CI: (0.27–0.77), *p* = 0.004), N1 single station vs. N0 (HR = 2.55, 95% CI: (1.33–4.90), *p* = 0.005), N1 multiple station vs. N0 (HR = 9.16, 95% CI: (3.65–23.0), *p* < 0.001), and N2 multiple stations vs. N0 (HR = 10.5, 95% CI: (3.44–32.2), *p* < 0.001); N2 single station was not a significant risk factor for relapse compared to N0 (HR = 1.82, 95% CI: (0.70–4.74), *p* = 0.11) (Table 3).

**Figure 1:**
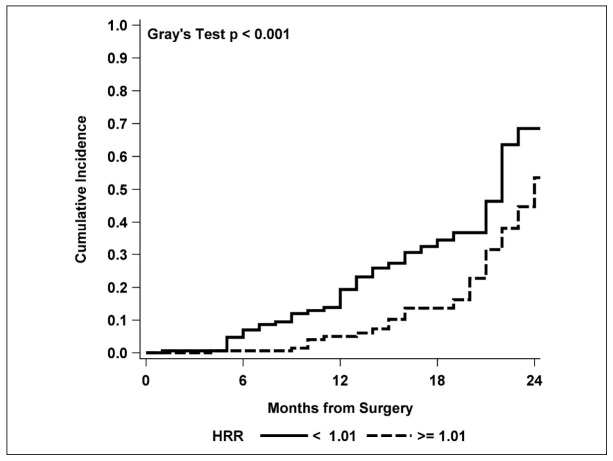
Cumulative incidence functions for relapse by the HRR median cut-off value.

Comparison of the cumulative incidence functions for relapse also showed a higher and significant increase with time for HRR values lower than 1.01 vs. HRR ≥1.01 (*p* < 0.001) (Figure 1).

Fifty-six patients (16.1%) had post-operative complications: 18 patients (5.1%) had grade I complications, 23 patients (6.6%) had grade II complications, 7 patients (2.0%) had grade III complications, 2 patients (0.5%) had grade IV complications, six patients (1.7%) had grade V complications—5 of them receiving right pneumonectomy, and 1 left pneumonectomy—and then died within 30 days of the operation or during hospitalization. Pre-operative HRR did not disclose any prognostic value either for any complications (*p* = 0.26) or for minor (*p* = 0.53) and major complications (*p* = 0.29). (Table 4).

## 4. Discussion

Complete blood count (CBC) is routinely performed in neoplastic patients before starting almost any kind of treatment. Several parameters tested in a standard CBC have recently been shown to have predictive value in many neoplastic diseases. These include Hb, RDW, neutrophil-to-lymphocyte ratio (NLR), and platelet-to-lymphocyte ratio (PLR) [18,19].

There is now clear evidence that the host response to systemic inflammation is closely related to tumor development and its progression; similarly, higher values of RDW—defined as anisocytosis—have been correlated to systemic inflammation and thus to an aggressive tumor behavior [20].

Nevertheless, although many studies disclosed interesting findings about the prognostic value of RDW in neoplastic disease, it has been postulated that RDW alone—without further indicators—might not completely represent the systematic inflammatory condition of the patient, in particular when chemotherapy has already been administered. For this reason, Sun et coll.—considering that Hb is a well-established marker of nutritional status—combined RDW and Hb values to build a new prognostic index for esophageal squamous cell carcinoma, disclosing a significant association between the HB/RDW ratio and clinical characteristics and survival outcomes in this cohort of patients [4].

Both RDW and Hb are influenced by many non-neoplastic conditions, and the Hb/RDW ratio is therefore a means of reducing the potential bias due to interfering factors, reflecting a more global health status of the patient. Our findings suggest that the HRR is an effective prognostic tool in terms of disease-free survival; in fact, considering the HRR as a categorical variable, patients with pre-operative HRR lower than 1.01 presented a higher risk for shorter disease-free survival, suggesting a stricter follow-up for patients with early-stage diseases or encouraging adjuvant treatments for those with tumors in more advanced stages. Similarly, considering HRR as a continuous variable, we observed recurrence risk reductions up to 85% for each unit of increase of the pre-operative HRR.

The HRR is a simple, inexpensive laboratory test included in standard CBC routinely performed before surgery; it does not require any additional procedure, cost, or dedicated equipment; its cost-effectiveness, considering its valuable prognostic efficacy, represents an added value of the test. Although we demonstrated a clear association only between preoperative HRR and DFS, we may assume that the HRR could be useful during the follow-up too. Further studies are required to explore this hypothesis.

As expected, our study disclosed a higher incidence of recurrence for patients with advanced-stage tumors and mediastinal involvement; interestingly, single-station-N2 disease did not show a significant risk increase (*p* = 0.12) that, instead, we observed in diseases with N1 multiple- (*p* < 0.001) and N2 multiple- (*p* < 0.001) station involvement. Although this may simply be due to a sampling effect, we could argue that single-station-N2 disease may represent a more favorable subgroup of patients, as reported in recent literature [21,22,23,24,25].

Finally, no difference was observed between open and minimally invasive approaches, thus confirming the now well-known concept that the minimally invasive approach is at least not inferior to open surgery in terms of oncological results; in any case, we adopted a minimally invasive approach frequently for patients with an early-stage disease and only in a few cases for carefully selected N2 patients [26,27].

Several limitations of the present study need to be considered: this is a single-center, non-randomized retrospective study, with a limited follow-up due to the only recent introduction of RDW in the standard pre-operative assessment of our patients. This conditioned only a small number of deaths, which were mostly due to post-operative major complications rather than to oncologic causes. For these reasons, we focused our analysis on disease-free survival rather than on overall survival (Table S1). Similarly, we did not explore the impact of pre-operative comorbidities on overall survival.

## 5. Conclusions

Pre-operative HRR is an effective prognostic factor of disease-free survival for resected-lung-adenocarcinoma patients; together with the number of pathologic node stations involved, it could therefore be considered as a further tool for planning adjuvant treatments and setting up a patient-tailored follow-up program.

## Figures and Tables

**Table 1:** Patients’ characteristics and treatments.

Characteristic	Level	All Patients ^a^ *n* = 342
Age at Surgery, years		66.0 (9.0)
	≤70	228 (66.7)
	>70	114 (33.3)
Tumor Size, mm		30.4 (19.7)
Hemoglobin (g/dL)		13.5 (1.6)
	Low (<11.8)	47 (13.7)
	Normal (11.8–15.8)	272 (79.5)
	High (>15.8)	23 (6.7)
RDW (%)		14.1 (2.0)
HRR ^b^		0.98 (0.19)
Female Gender		163 (47.1)
Smoker	Yes	264 (77.2)
	No	75 (21.9)
	missing	3 (0.9)
pN	N0	204 (59.7)
	N1	67 (19.6)
	Single station	55 (16.1)
	Multiple station	12 (3.5)
	N2	71 (20.8)
	Single station	54 (15.8)
	Multiple station	17 (5.0)
N Status (*n* = 138) ^c^	Single station	109 (79.0)
	Multiple stations	29 (21.0)
Pathological Stage	pT1	136 (39.8)
	pT2	141 (41.2)
	pT3	46 (13.5)
	pT4	19 (5.6)
Stage	1A,1B	169 (49.4)
	2A,2B	71 (20.8)
	3A,3B	102 (29.8)
Grading	0	22 (6.4)
	1	20 (5.9)
	2	159 (46.5)
	3	112 (32.8)
	missing	29 (8.5)
Surgery	Open	188 (54.8)
	Minimal Invasive Surgery	154 (45.2)
Adjuvant Treatments	Yes	93 (27.2)
	unknown	6 (1.8)
Neoadjuvant Treatments	Yes	54 (15.8)
	unknown	4 (1.2)
Any treatment	Yes	121 (35.4)
	unknown	4 (1.2)
Procedures ^d^	Lobectomy	318 (93.0)
	Pneumonectomy	24 (7.0)

^a^ Statistics are means (SD) for: age, tumor size, hemoglobin, red cell distribution width (RDW), HRR; N (column%) otherwise; ^b^ HRR = hemoglobin/RDW ratio; ^c^ N1 and N2 patients subgroup only; ^d^ See text for details.

**Table 2:** Disease-free survival univariate analysis.

Risk Factor at Surgery		Events/At Risk	HR (95% CI)	*p*-Value
Age		65/342	1.17 ^a^ (0.99–1.38)	0.07
Tumor Size		65/342	1.21 ^b^ (1.04–1.40)	0.01
Hemoglobin		65/342	0.84 ^c^ (0.72–0.97)	0.02
RDW%		65/342	1.16 ^c^ (1.05–1.28)	0.003
HRR				
Continuous		65/342	0.15 ^c^ (0.05–0.50)	0.002
Median cut-off	<1.01	44/170	1	
	≥1.01	21/172	0.41 (0.25–0.68)	<0.001
pN	N0	23/204	1	
	N1 Single Station	15/55	3.30 (1.78–6.11)	<0.001
	N1 Multiple Stations	5/12	6.47 (2.37–17.7)	<0.001
	N2 Single Station	12/54	1.79 (0.87–3.67)	0.12
	N2 Multiple Stations	19/17	9.25 (3.57–24.0)	<0.001
N Status ^d^	Single station	27/109	1	
	Multiple stations	15/29	2.84 (1.43–5.63)	0.003
Gender	Female	27/161	1	
	Male	38/181	1.23 (0.76–1.98)	0.41
Smoker	No	12/75	1	
	Yes	53/264	0.85 (0.49–1.49)	0.58
Surgery	Open	42/188	1	
	Minimal Invasive Surgery	23/154	0.66 (0.41–1.06)	0.08
pN	N0	23/204	1	
	N1	20/67	3.69 (2.10–6.48)	<0.001
	N2	22/71	2.85 (1.55–5.27)	<0.001
pT	pT1	12/136	1	
	pT2	35/141	2.22 (1.18–4.18)	0.01
	pT3	13/46	3.01 (1.35–6.71)	0.007
	pT4	5/19	3.33 (0.99–11.2)	0.05
Grading	0	4/22	1	
	1	0/20	Not estimable	-
	2	22/159	0.74 (0.26–2.16)	0.58
	3	30/112	1.80 (0.62–5.25)	0.28
Stage	1A,1B	16/169	1	
	2A,2B	17/71	2.00 (1.08–3.69)	0.03
	3A,3B	32/102	3.72 (2.06–6.69)	<0.001
Treatments	No	32/217	1	
	Yes	33/121	1.67 (1.02–2.73)	0.04
Procedure	Lobectomy	60/318	1	
	Pneumonectomy	5/24	1.99 (0.74–5.37)	0.17

^a^ by five-year increase; ^b^ by 10 mm increase; ^c^ by one unit increase; ^d^ N1 and N2 patients subgroup only; median follow-up = 13 months.

**Table 3:** Disease-free survival multivariable analysis treatment-adjusted.

Risk Factor at Surgery		HR (95% CI)	*p*-Value
Tumor Size		1.18 ^a^ (0.99–1.40)	0.06
HRR Median cut-off	≥1.01	1	
	<1.01	2.20 (1.30–3.72)	0.004
pN	N0	1	
	N1 Single Station	2.55 (1.33–4.90)	0.005
	N1 Multiple Stations	9.16 (3.65–23.0)	<0.001
	N2 Single Station	2.29 (0.83–6.33)	0.11
	N2 Multiple Stations	10.5 (3.44–32.2)	<0.001

^a^ by 10 mm-unit increase; median follow-up = 13 months.

**Table 4:** Estimates of the risk for major, minor, and any grade complications (odd ratios) according to the HRR median cut-off.

Complication	HRR	OR (95% CI)	*p*-Value
Major	<1.01	1	
	≥1.01	1.82 (0.60–5.55)	0.29
Minor	<1.01	1	
	≥1.01	1.24 (0.64–2.40)	0.53
Any complications	<1.01	1	
	≥1.01	1.41 (0.78–2.53)	0.26

## Data Availability

Available on request.

## Supplementary Materials

The following are available online at https://www.mdpi.com/2072-6694/13/4/710/s1, Table S1: All multivariable subdistribution Hazard Ratios (sHR) for treatment adjusted Disease Free Survival.
