# Supplementary material for: Prognostic Value of the Hemoglobin/Red Cell Distribution Width Ratio in Resected Lung Adenocarcinoma
Source: Cancers (Basel). 2021 Feb 9;13(4):710. doi: 10.3390/cancers13040710 (PMC7916257; doi:10.3390/cancers13040710)
Supplement: Supplementary file 1 [file cancers-13-00710-s001.pdf]

Table S1. All multivariable subdistribution Hazard Ratios (sHR) for treatment adjusted Disease Free Survival

**Risk Factor at Surgery**		**sHR (95% CI)**
Tumor Size		1.18[a] (0.99 -1.40)
HRR Median cut-off	≥ 1.01	1
	< 1.01	2.20 (1.30-3.72)
	< 1.01	1
	≥ 1.01	0.46 (0.27-0.77)
pN	N0 vs N1 multiple	0.11 (0.04-0.27)
	N1 multiple vs N0	9.16 (3.65-23.0)
	N0 vs N1 single	0.39 (0.20-0.75)
	N1 single vs N0	2.55 (1.33-4-90)
	N0 vs N2 multiple	0.10 (0.03-0.29)
	N2 multiple vs N0	10.5 (3.43-32.2)
	N0 vs N2 single	0.44 (0.16-1.20)
	N2 single vs N0	2.29 (0.83-6.33)
	N1 multiple vs N1 single	3.59 (1.38-9.35)
	N1 single vs N1 multiple	0.28 (0.11-0.72)
	N1 multiple vs N2 multiple	0.87 (0.27-2.81)
	N2 multiple vs N1 multiple	1.15 (0.36-3.70)
	N2 multiple vs N2 single	4.00 (1.39-11.5)
	N2 single vs N1 multiple	0.25 (0.09-0.72)
	N1 single vs N2 multiple	0.24 (0.08-0.74)
	N2 multiple vs N1 single	4.13 (1.35-12.7)
	N1 single vs N2 single	1.11 (0.42-2.98)
	N2 single vs N1 single	0.90 (0.34-2.41)
	N2 multiple vs N2 single	4.59 (1.52-13.8)
	N2 single vs N2 multiple	0.22 (0.07-0.66)

[a] by 10 mm units increase; Median Follow-up = 13 months
